# CAG Repeats Within the Non-pathological Range in the HTT Gene Influence Personality Traits in Patients With Subjective Cognitive Decline: A 13-Year Follow-Up Study

**DOI:** 10.3389/fpsyt.2022.826135

**Published:** 2022-03-11

**Authors:** Valentina Moschini, Salvatore Mazzeo, Silvia Bagnoli, Sonia Padiglioni, Filippo Emiliani, Giulia Giacomucci, Carmen Morinelli, Assunta Ingannato, Tommaso Freni, Laura Belloni, Camilla Ferrari, Sandro Sorbi, Benedetta Nacmias, Valentina Bessi

**Affiliations:** ^1^Strutture Organizzative Dipartimentali Neurologia 1, Dipartimento Neuromuscolo-Scheletrico e Degli Organi di Senso, Azienda Ospedaliero Universitaria Careggi, Florence, Italy; ^2^Department of Neuroscience, Psychology, Drug Research and Child Health (NEUROFARBA), University of Florence, Florence, Italy; ^3^Istituto di Ricovero e Cura a Carattere Scientifico Fondazione Don Carlo Gnocchi, Florence, Italy; ^4^Regional Referral Centre for Relational Criticalities, Florence, Italy; ^5^Unit Clinic of Organizations Careggi University Hospital, Florence, Italy

**Keywords:** subjective cognitive decline (SCD), mild cognitive impairment (MCI), Huntington (disease), Alzheimer's disease, HTT CAG repeat, personality, intermediate alleles, big five questionnaire

## Abstract

**Objective:**

*HTT* is a gene containing a key region of CAG repeats. When expanded beyond 39 repeats, Huntington disease (HD) develops. *HTT* genes with <35 repeats are not associated with HD. The biological function of CAG repeat expansion below the non-pathological threshold is not well understood. In fact higher number of repeats in HTT confer advantageous changes in brain structure and general intelligence, but several studies focused on establishing the association between CAG expansions and susceptibility to psychiatric disturbances and to other neurodegenerative disease than HD. We hypothesized that *HTT* CAG repeat length below the pathological threshold might influence mood and personality traits in a longitudinal sample of individuals with Subjective Cognitive Decline.

**Methods:**

We included 54 patients with SCD. All patients underwent an extensive neuropsychological battery at baseline, *APOE* genotyping and analysis of *HTT* alleles. We used the Big Five Factors Questionnaire (BFFQ) and Hamilton Depression Rating Scale (HDRS), respectively, to assess personality traits of patients and depression at baseline. Patients who did not progress to Mild Cognitive Impairment (MCI) had at least 5-year follow-up time.

**Results:**

In the whole sample, CAG repeat number in the shorter *HTT* allele was inversely correlated with conscientiousness (Pearson = −0.364, *p* = 0.007). There was no correlation between HDRS and CAG repeats. During the follow-up, 14 patients [25.93% (95% C.I. = 14.24–37.61)] progressed to MCI (MCI^+^) and 40 [74.07% (95% C.I. = 62.39–85.76)] did not (MCI^−^). When we performed the same analysis in the MCI^+^ group we found that: CAG repeat length on the shorter allele was inversely correlated with energy (Pearson = 0.639, *p* = 0.014) and conscientiousness (Pearson = −0.695, *p* = 0.006). CAG repeat length on the longer allele was inversely correlated with conscientiousness (Pearson = −0.901, *p* < 0.001) and directly correlated with emotional stability (Pearson = 0.639, *p* = 0.014). These associations were confirmed also by multivariate analysis. We found no correlations between BFFQ parameters and CAG repeats in the MCI^−^ group.

**Discussion:**

Personality traits and CAG repeat length in the intermediate range have been associated with progression of cognitive decline and neuropathological findings consistent with AD. We showed that CAG repeat lengths in the *HTT* gene within the non-pathological range influence personality traits.

## Introduction

Huntingtin (*HTT*) is a gene coding for a soluble peptide which is widely expressed during development, being essential for embryogenesis (1), and plays crucial roles in axonal trafficking, regulation of gene transcription, and cell survival in post developmental life (2). *HTT* contains a key region of CAG repeats which is translated into a corresponding polyglutamine stretch (3). The expansion of CAG triplet beyond 40 repeats leads to the dysfunction and death of neurons in the striatum and in other brain regions, causing Huntington's disease (HD) (4), a neurodegenerative disorder characterized by cognitive, motor and psychiatric disturbance (5).

Several studies showed that carriers of *HTT* CAG repeats in the pathological range (HD carriers) had a defined personality profile, characterized by higher conscientiousness, lower emotional stability (6) and lower social cognition (7) as compared to controls.

However, there are only a few studies exploring the effect of CAG repeats in the non-pathological range on personality traits and mood. Killoran et al. showed that the individuals with a number of CAG repeats in the range of the so-called intermediate alleles (IA) (27–35 CAG repeats) were more likely to experience trouble with motivation, to have thoughts of suicide and generally reported more mood and behavior problems than those with <27 repeats (8). Other authors showed that IAs of *HTT* are associated with higher prevalence of neurodegenerative conditions, such as Alzheimer's disease (AD) (9) and to the non-fluent variant of primary progressive aphasia (10). IA proportion has been reported to be significantly increased in AD patients (8) as compared to healthy controls and *HTT* levels were increased in neuronal cells in the hippocampus of AD cases (11). Alzheimer-type lesions, in turns, were found more frequently in autopsy of HD patients [from 67 (12) to 80% (13)] as compared to healthy controls. Finally, a recent work by our group found that *HTT* CAG expansions influence neuropsychological functions in individuals experiencing Subjective Cognitive Decline (SCD) or Mild Cognitive Impairment (MCI) (14).

Therefore, in the present study we hypothesized that *HTT* CAG repeat length below the pathological threshold might influence mood and personality traits in a longitudinal sample of individuals with SCD.

## Materials and Methods

### Participants and Clinical Assessment

We included 54 consecutive patients who complained memory disorder and self-referred to the Center for Alzheimer's disease and Adult Cognitive Disorders of Careggi Hospital in Florence. Inclusion criteria were: (1) complaining of cognitive decline with a duration of ≥ 6 months; (2) normal functioning on the Activities of Daily Living and the Instrumental Activities of Daily Living scales (15); (3) unsatisfied criteria for dementia or MCI at baseline (16, 17). Exclusion criteria were: (1) history of head injury, current neurological and/or systemic disease, symptoms of psychosis, major depression, alcoholism or other substance abuse. No patient had family history of Huntington disease.

The local ethics committee approved the protocol of the study. All participants gave written informed consent. All procedures involving experiments on humans have been done in accordance with the ethical standards of the Committee on Human Experimentation of the institution in which the experiments were done or in accordance with the Helsinki Declaration of 1975. Specific national laws have been observed.

At baseline, all participants underwent: (1) comprehensive family and clinical history, general and neurological examination; (2) extensive neuropsychological battery including assessment of cognitive complaints, depressive symptoms, and premorbid intelligence; (3) brain MRI or CT scan; (4) peripheral blood collection to analyze Apolipoprotein E (*APOE*) and *HTT* genotypes. A positive family history of dementia was defined as one or more first-degree relatives with documented cognitive decline. Disease duration was defined as the time from the onset of symptoms to the first neurological or neuropsychological evaluation.

All patients underwent clinical and neuropsychological follow-up every 12 or 24 months. Progression to MCI and conversion to AD were defined according to the National Institute on Aging-Alzheimer's Association (16, 17).

### Neuropsychological Assessment

All patients underwent an extensive neuropsychological assessment, consisting of: global measurements (Mini-Mental State Examination), tasks exploring verbal and spatial working memory (Digit Span; Corsi Tapping Test), verbal long-term memory (Five Words and Paired Words Acquisition; Recall after 10 min; Recall after 24 h; 15 Words of Rey Babcock Short Story Immediate and Delayed Recall), language [Token Test; Category Fluency Task, Phonemic Fluency Test (15)],visual-spatial abilities (Rey-Osterrieth Complex Figure copy), visual-spatial long-term memory [Rey-Osterrieth Complex Figure test (18)], attention/executive function [Dual Task (19), and Trail Making Test (20)], everyday memory (Rivermead Behavioral Memory Test) (18). All raw test scores were adjusted for age, education and gender according to the correction factor reported in validation studies for the Italian population (15, 18–22).

Cognitive complaints were explored using a survey based on the Memory Assessment Clinics-Questionnaire (MAC-Q) (23).The presence and severity of depressive symptoms was evaluated by the 22-item Hamilton Depression Rating Scale (HRSD) (24). Premorbid intelligence, as a cognitive reserve proxy, was assessed at by *Test di Intelligenza Breve* (TIB, i.e., Brief Intelligence Test) (25), an Italian version of the National Adult Reading Test (NART) (26).

### Personality Traits

Personality traits were assessed by the Big Five Factors Questionnaire (BFFQ), a self-administered inventory following a widely accepted five-traits personality model (27). The five dimensions of the BFFQ are: (1) emotional stability: the resilience to unpleasant emotions like anger, anxiety, depression, self-pity and worry; (2) energy: being active, assertive, energetic, enthusiastic, outgoing, and talkative; (3) conscientiousness: the degree of organization, persistence, and motivation in goal-directed behavior; (4) agreeableness: the quality of interpersonal orientation to compassion, including adjectives like appreciative, forgiving, generous, kind, sympathetic, and trusting; (5) openness to culture and experience: active imaginations, aesthetic sensitivity, intellectual curiosity, wide variety of interests. Each of the five dimensions of personality consists of two sub-dimensions defined in turn by 24 items. Subjects rated their level of agreement on a five-point scale ranging from “strongly agree” to “strongly disagree”.

### *HTT* and *APOE* Genotyping

Subjects' DNA was isolated from peripheral blood using a standard automated method (QIAcube, QIAGEN). *APOE* genotypes were investigated by High Resolution melting Analyses (HRMA) (28). Two sets of PCR primers were designed to amplify *APOE* regions encompassing rs7412 [NC_000019.9:g.45412079C>T] and rs429358 (NC_000019.9:g.45411941T>C). Patients who were carriers of the ε4 allele (one or two *APOE*ε4 alleles) were classified as *APOE*ε4^+^, while patients who were not carriers of ε4 allele (no *APOE*ε4 alleles) were classified as *APOE*ε4^−^.

*HTT* CAG repeat expansion was determined by a polymerase chain reaction amplification assay, using fluorescently labeled primers (29). The size of the fragment was determined by capillary electrophoresis using SeqStudio Genetic Analyzer (ThermoFisher) and the GeneMapper version 4.0 software (Applied Biosystems). A set of *HTT* CAG alleles, whose lengths were confirmed by DNA sequencing, was used to provide size standards. Patients who were carriers of the intermediate allele [at least one *HTT* allele with CAG-repeat sizes of 27–35 repeats (5)] were classified as IA^+^, while patients who were not carriers were classified as IA^−^.

### Statistical Analysis

We tested for normality by the Shapiro-Wilk test. Patient groups were characterized by using means and standard deviations, median and interquartile range (IQR), frequencies or percentages and 95% confidence interval (95%C.I.) for continuous distributed variables, continuous non-normally distributed variables and categorical variables, respectively. Depending on the distribution of our data, we used *t*-test or non-parametric Mann-Whitney-U Tests for between-groups comparisons, Pearson's correlation coefficient or non-parametric Spearman's ρ (rho) to evaluate correlations between groups' numeric measures, and chi-square tests to compare categorical data. We calculated the size effect by Cohen's *d* for normally distributed numeric measures, η^2^ for Mann-Whitney-U Test and Cramer's *V* for categorical data. We used backward linear regressions as multivariate analyses. Bonferroni correction was applied to correct for multiple comparisons dividing 0.05 by the numbers of variables included in each analysis (adjusted statistical significance levels are reported in the caption of each table). All statistical analyses were performed with SPSS software v.25 (SPSS Inc., Chicago, USA) and the computing environment R 4.0.3 (R Foundation for Statistical Computing, Vienna, 2013).

## Results

### Demographic and Clinical Features at Baseline, Frequency Distributions of CAG Repeats and Comparison Between IAs^–^ and IAs^+^

**Figure 1:**
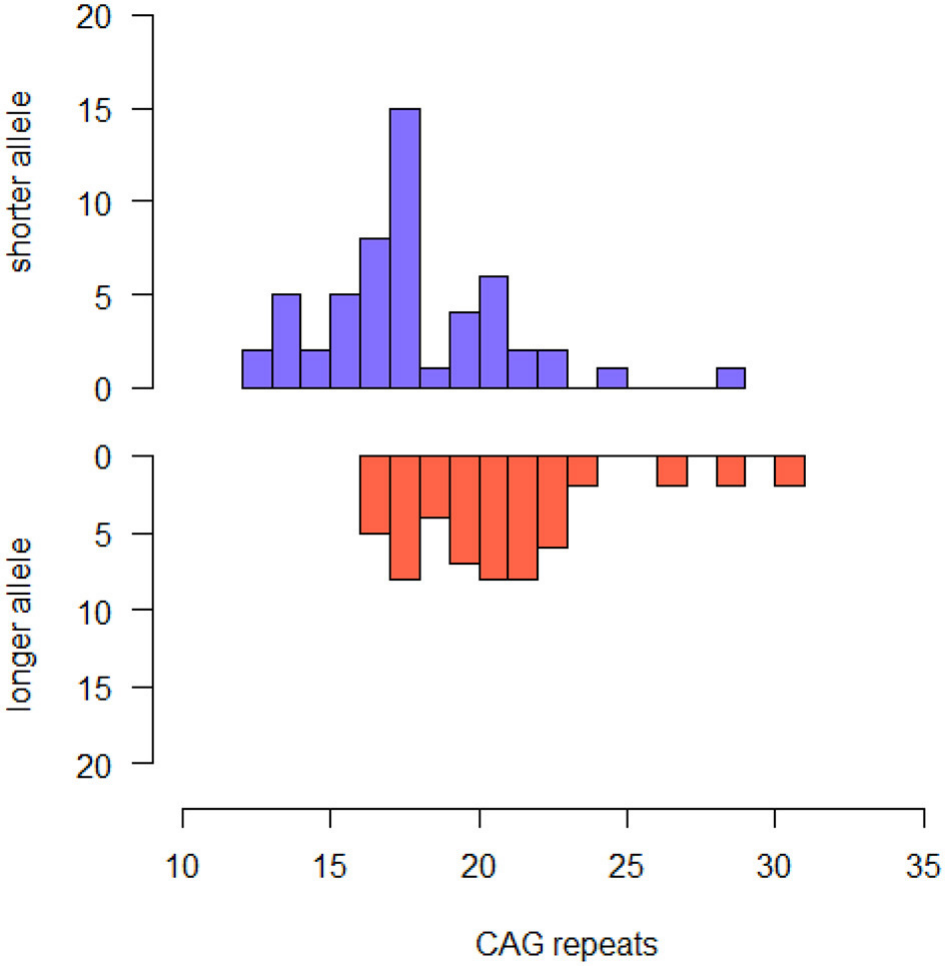
Histogram describing the frequency of CAG repeat lengths of shorter and longer alleles in the whole sample.

Our sample included 37 male and 13 females. Mean age at baseline was 60.80 (± 7.33). All the patients were Caucasian. Median CAG repeats lengths were 18.00 (IQR 4.00, range: 12–29) in the shorter allele and 21.00 (4.00, range: 16–31) in the longer allele. The most common *HTT* alleles had 18 (shorter alleles) and 18, 21 and 22 (longer alleles) CAG-repeats (Figure 1). Six out of 54 patients [11.11% (95% CI 2.73–19.49)] were carriers of intermediate alleles of *HTT* gene (IAs^+^). Among these, one patient was homozygous for IA (29 and 31 CAG-repeats). There was no significant difference in disease duration, family history of AD, sex, years of education, TIB, MMSE, HDRS and BFFQ score between IAs^−^ and IAs^+^. There was no difference in any neuropsychological test score between IAs^−^ and IAs+.

Patients were followed-up for a median time of 13 years. During the follow-up, 14 patients [25.93% (95% C.I. = 14.24–37.61)] progressed to MCI (MCI^+^) and 40 patients [74.07% (95% C.I. = 62.39–85.76)] did not progress to MCI (MCI^−^) with a mean follow-up time of 15.29 (IQR 4.44). Follow-up time of MCI^−^was significantly longer than mean progression time of MCI^+^ [8.35 (IQR 6.33), *p* < 0.001, η^2^ = 0.27] years. There was no difference in proportion of IA between MCI^−^and MCI^+^ (*p* = 0.661).

At baseline no significant differences were found with respect to disease duration, family history of AD, sex, years of education, TIB, MMSE, HDRS and BFFQ score between MCI^−^and MCI^+^ (Table 1).

**Table 1:** Baseline features in the whole group and comparison between MCI^−^ and MCI^+^.

	**SCD**	**MCI^−^**	**MCI^+^**
*N* [% (95% C.I.)]	54	40 [74.07 (62.39–85.76)]	14 (25.93 [14.24–37.61])
Age at baseline, mean (SD)	60.80 (7.33)	59.99 (7.63)	63.11 (6.073)
Age at onset, mean (SD)	56.13 (9.09)	54.73 (9.67)	60.14 (5.75)
Disease duration, median (IQR)	3.33 (5.09)	3.58 (4.02)	2.72 (1.30)
Follow-up time, median (IQR)	15.29 (4.67)	15.29 (4.44)^***^	8.35 (6.33)^***^
Sex (women/men)	37/13	26/14	11/3
Family history of dementia, % (95% C.I.)	51.85% (38.52–65.18)	52.50% (37.02–67.98)	50.00% (23.81–76.19)
Years of education, median (IQR)	12.50 (8)	12.58 (4.41)	11 (5.97)
TIB, median (IQR)	113.09 (4.57)	113.11 (4.49)	113.04 (20.29)
CAG repeat (shorter allele), median(IQR)	18.00 (4)	18.00 (4)	17.50 (4)
CAG repeat (longer allele), median(IQR)	21.00 (4)	21.00 (4)	19.50 (7)
*APOE* ε4^+^, % (95% C.I.)	31.71% (17.46–45.95)	17.50% (5.72–29.28)	42.86% (16.93–68.78)
IA^+^ % (95% C.I.)	11.11% (2.73–19.49)	66.67% (28.95–104.39)	33.33% (−4.39–71.05)
MMSE, median (IQR)	29.00 (1)	30.00 (1)	29.00 (1)
HDRS, median (IQR)	25.00 (6.00)	25.00 (6.00)	26.50 (7.75)
MAC-Q, mean (SD)	25.84 (2.57)	25.84 (2.54)	25.81 (2.79)
Energy, mean (SD)	45.50 (10.24)	46.55 (10.42)	42.50 (9.42)
Friendship, mean (SD)	49.80 (9.12)	48.80 (9.27)	52.64 (8.33)
Conscientiousness, mean (SD)	46.83 (9.86)	45.75 (10.24)	49.93 (8.23)
Emotional Stability, mean (SD)	49.28 (10.52)	48.05 (10.04)	52.79 (11.45)
Openness, mean (SD)	46.91 (10.41)	47.20 (10.34)	46.07 (10.80)

*Demographic data. Values quoted in the table are mean (SD), median (IQR), percentages (95% C.I.), or frequencies. Age at baseline, age at onset, follow-up time, disease duration are expressed in years. Statistical significance received a Bonferroni adjustment and being accepted at the p < 0.003 and p < 0.016 for categorical variables*

(^***^p < 0.001, η^2^ = 0.27).

*TIB: MMSE, Mini Mental State Examination; HDRS, Hamilton Depression Rating Scale; MAC-Q, Memory Assessment Clinics-Questionnaire*.

### Correlations Between Personality Traits, Demographic and Genetic Features

In the whole sample, energy was directly correlated with conscientiousness (Pearson = 0.318, *p* = 0.019) and openness (Pearson = 0.614, *p* < 0.001). Emotional stability was directly correlated with conscientiousness (Pearson = 0.357, *p* = 0.008) and inversely correlated with HDRS (Pearson = −0.30, *p* = 0.028). Openness was significantly higher in man than in women (51.47 ± 10.51 vs. 4481 ± 9.80, *p* = 0.028, Cohen's *d* = 0.66). BFFQ scores were not associated with demographic features, neuropsychological scores and *APOE* genotype.

CAG repeat number in the shorter allele was inversely correlated with conscientiousness (Pearson = −0.364, *p* = 0.007). There was no correlation between CAG repeat numbers and HDRS scores.

**Figure 2:**
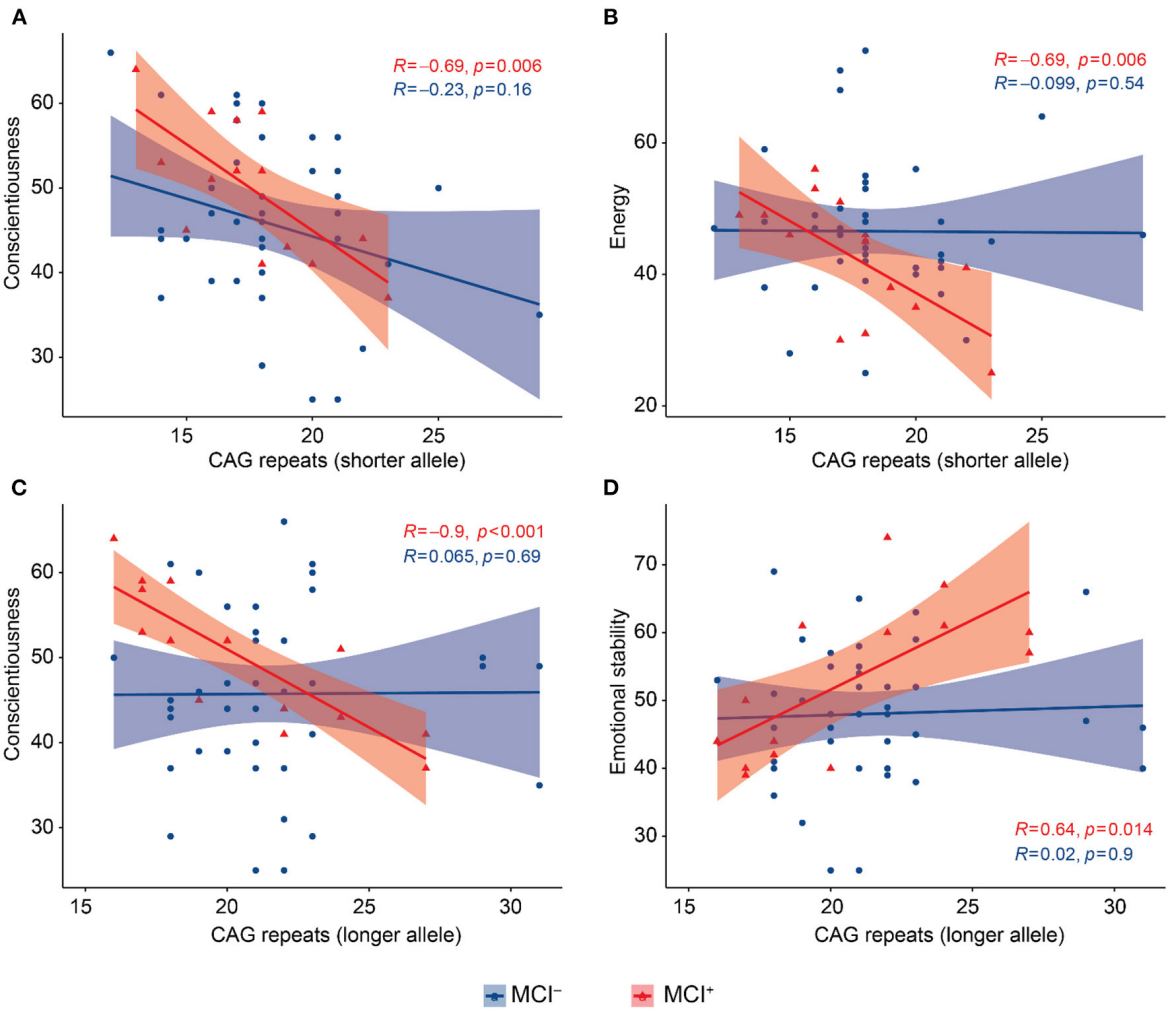
Scatter plots with lines of best fit (95% C.I.) showing the relationship between CAG repeat lengths and BFFQ dimensions in MCI^−^ and MCI^+^. Pearson correlation coefficient (R) and level of significance (p) are reported (statistical significance at the *p* < 0.05). **(A)** CAG repeat length on the shorter allele was inversely correlated with conscientiousness in MCI^+^. **(B)** CAG repeat length on the shorter allele was inversely correlated with energy in MCI^+^. **(C)** CAG repeat length on the longer allele was inversely correlated with conscientiousness in MCI^+^. **(D)** CAG repeat length on the longer allele was directly correlated with emotional stability in MCI^+^.

We performed this analysis in the MCI^−^ and MCI^+^ groups separately. In the MCI^+^ group CAG-repeat length on the shorter allele was inversely correlated with conscientiousness (Pearson = −0.695, *p* = 0.006, Figure 2A) and energy (Pearson = −0.694 *p* = 0.006, Figure 2B); CAG repeat length on the longer allele was inversely correlated with conscientiousness (Pearson = −0.901, *p* < 0.001, Figure 2C) and directly correlated with emotional stability (Pearson = 0.639, *p* = 0.014, Figure 2D). We found no correlations between BFFQ parameters and CAG repeats in the MCI^−^ group. There was no association between HDRS score and CAG repeat length neither in MCI^−^ nor in MCI^+^ group.

### Multivariate Analysis and Regression Models

To ascertain that the associations between personality traits and CAG repeat lengths were independent from confounding factors, we performed multiple regression analyses in the MCI^+^ group. Energy, emotional stability and conscientiousness were considered as dependent variables one at a time. CAG repeat numbers in the shorter and longer allele were both considered as independent variables. Variables that have been found to be associated with personality traits have been considered as covariates. There was no evidence of multicollinearity among covariates, as assessed by tolerance values >0.10. Energy remains significantly associated with CAG repeat length on the *HTT* shorter allele [*B* = −2.33 (95%CI = −4.02:−6.4), *p* = 0.011]; emotional stability remains significantly associated with CAG repeats on the *HTT* longer allele [*B* = 2.30 (95%CI = 1.26:3.34), *p* = 0.001] and with HDRS [*B* = −1.49 (95%CI = −2.24:−0.73), *p* = 0.001]; conscientiousness remains significantly associated with *HTT* longer allele [*B* = −1.46 (95%CI = −2.26:−0.66), *p* = 0.002] (Table 2).

**Table 2:** Multiple regression model.

**Energy**	***B***	**95% C.I. for B**		**β**	***P***
		**Lower**	**Upper**		
(Constant)	83.42	53.36	113.49	–	<0.001
***HTT*** **shorter**	**−2.33**	**−4.02**	**−6.4**	**−0.66**	**0.011**
**Conscientiousness**					
(Constant)	100.32	82.35	118.281	–	<0.001
*HTT* shorter	−1.07	−2.19	−0.05	−0.34	0.059
***HTT*** **longer**	**−1.46**	**−2.26**	**−0.66**	**−0.66**	**0.002**
**Emotional stability**					
*HTT* shorter	1.35	−0.09	2.78	0.45	0.063
***HTT*** **longer**	**2.30**	**1.26**	**3.34**	**0.94**	**0.001**
**HDRS**	**−1.49**	**−2.24**	**−0.73**	**−0.74**	**0.001**
Conscientiousness	0.368	0.07	0.66	0.34	0.020

*Unstandardized Regression Coefficients (B) and 95% Confidence Intervals (95% C.I.), standardized coefficient (β) and p-value (p), are reported. Statistical significance received a Bonferroni adjustment and was accepted at the p < 0.012 (in bold characters)*.

### Discussion

In the present study we explored the association between CAG repeat lengths in the *HTT* gene with personality traits and mood in a sample of SCD patients followed-up for a median time of 13 years. As the main result we found that the length of CAG repeats in the *HTT* gene within the non-pathological range are associated with personality traits in SCD patients who progressed to MCI but not in patients who remained stable. In particular, lower level of conscientiousness and energy and higher level of emotional stability were correlated with higher number of CAG repeats. These associations were independent from possible confounding variables and were not influenced by depressive symptoms measured by HDRS. An association between *HTT* variants and personality traits was shown by Larsen et al. (6). Interestingly, these authors found opposite associations: HD carriers and healthy first-degree relatives (individuals at risk for HD) had greater conscientiousness and lower emotional stability as compared to controls. However, they did not find any difference between HD carriers and non-carriers at risk for HD, concluding that there is no direct effect of the *HTT* gene on personality traits. The discrepancies with our results could be due to the different populations considered. Larsen et al. enrolled both HD carriers and HD non-carriers with family history for HD while none of the individuals included in our sample had family history for HD. It is known that HD symptoms and onset are influenced also by modifier genes other than *HTT* (30, 31). Therefore, we could speculate the opposite effect of *HTT* in individuals at risk and not at risk for HD might follow a polygenic effect.

Moreover, Larsen et al. did not report CAG repeat numbers of the control group. So, we do not know if the effect on personality traits is due to the higher number of CAG repeats or to the family history for HD. We aim to test these hypotheses in future studies on larger samples.

To the best of our knowledge there are no previous reports showing an effect of *HTT* CAG repeat length on personality traits in non-HD patients. Even more importantly, our results showed that his effect may depend on the pathological background of the SCD.

SCD is an heterogeneous condition including normal aging, psychiatric conditions, neurologic and medical disorders, substance use, and medication (32). Longitudinal data allowed us to isolate patients whose memory symptoms were more probably due to a neurodegenerative condition underlying SCD. In other words, it is probable that the SCD of patients who progressed to MCI was due to a degenerative disease already active at the SCD stage.

We already showed that CAG repeat lengths in *HTT* may differently influence neuropsychological performances according to cognitive status of subjects (14). In more detail, we showed that a higher number of GAC repeats in the non-pathological range was associated with higher scores in tasks assessing executive function, memory, visual–spatial ability and language in SCD patients but to lower scores in the same cognitive domain in MCIs. Therefore, we might speculate that the effect found in this study of *HTT* variants both on cognitive function and on personality traits may be evident only when a pathological process occurs. In other words, as mutant *HTT* leads to a defined personality profile in HD patients, the number of CAG repeats in *HTT* influence personality traits and neuropsychological function also in patients with a probable neurodegenerative process underlying a subjective or objective cognitive decline.

The biological substrate of this effect might lie in the interaction of *HTT* protein with a number of proteins with a role in microtubule-based axon trafficking (33, 34). In particular, wild-type Hintingtin protein specifically enhances the vesicular transport of Brain Derived Natriuretic Factor (BDNF) (35), a neurotrophic factor involved in synaptic connections (36), neural growth (37), synaptic plasticity (38), and essential for long-term potentiation underlying hippocampus-related memory (14, 39). PolyQ tracts in Huntingtin protein stabilize interactions (40), according to a non-linear relation with the best function reached at an intermediate number of CAG repeats and then showing a progressive decrease (41). We might speculate that the interaction between Huntingtin and BDNF or the effect of this interaction on axonal trafficking may depend not only on the length of PolyQ tracts, but also on a possible pathological process underlying the progression from SCD to MCI. We think that our results could represent first clinical evidence to further explore this mechanism.

It would be interesting to know if this effect changes over time, for instance, collecting BFFQ data also at the end of the follow-up and in patients without SCD.

The relationships of personality traits with SCD and risk of progression of cognitive decline have been already reported by previous works with different results. Lower conscientiousness has been associated with higher complaints about their memory in SCD (42). Prospective studies indicate that individuals who score higher on conscientiousness have a slower rate of cognitive decline and reduced risk of developing dementia, even in the presence of AD neuropathology (43, 44). Conscientiousness is even associated with less amyloid deposition in cognitively normal aging (45). Low emotional stability has been reported as a risk factor for clinical AD and memory deficit has been associated with a faster rate of cognitive decline (46), while higher emotional stability seems to moderate the effect of the *APOE*ε4 on cognitive function and risk of dementia (47). On the contrary, in a previous study we showed that higher emotional stability was linked to higher risk of progression from SCD to MCI.

We are not aware of studies showing association of energy with SCD and progression to objective cognitive impairment. Nevertheless, it is well recognized that patients with AD dementia showed a reduction of social life, tendency to social isolation, loss of motivation or reduced initiative (48).

Interestingly, recent studies showed that carriers of IAs had higher β-amyloid burden and developed MCI more frequently than non-carriers of IAs (49).

Taking this evidence together, we could speculate that CAG repeat number in *HTT* gene might be one of the genetic factors mediating the effect of conscientiousness, emotional stability and energy on neurodegeneration and progression of cognitive decline. We aim to better explore this hypothesis in further studies, first of all expanding our sample size. Indeed, the small sample size is the first limitation of the present work, in particular when we classified patients according to the progression to MCI (only 14 patients were MCI^+^). Moreover, only six patients were carriers of the IA. A larger sample would allow us to assess for personality trait differences between IA^−^ and IA^+^. Another limitation is the lack of a control group. Finally, as it is a single-center study, there may be biases regarding assessment and diagnosis procedures and inclusion of only Caucasian participants.

However, this study has some remarkable strengths: this is the first study assessing the correlation of CAG repeat lengths with personality traits in patients without family history of HD. This may lead to the exclusion of a polygenic effect of modifier genes which are involved in HD symptoms and onset in individuals with family history for HD. The second strength is the very long, median follow-up time. In fact, follow-up time in the MCI^−^is much longer than the time of conversion of MCI^+^. This information allows us to minimize the possible underestimation of progression to MCI.

### Conclusions

We showed that length of CAG repeats in the *HTT* gene within the non-pathological range might influence personality traits in SCD patients who will progress to MCI. Low level of conscientiousness, low level of energy and high level of emotional stability are associated with a higher number of CAG repeats. Both personality traits and CAG repeat length in the intermediate range have been associated with progression of cognitive decline and neuropathological findings consistent with AD. If confirmed by further studies on larger samples, our results may be the key to reveal the missing link among *HTT* gene, personality traits and neurodegenerative process.

## Data Availability Statement

The original contributions presented in the study are included in the article/supplementary materials, further inquiries can be directed to the corresponding author.

## Ethics Statement

The studies involving human participants were reviewed and approved by Comitato Etico Regionale per la Sperimentazione Clinica della Regione Toscana. The patients/participants provided their written informed consent to participate in this study.

## Author Contributions

SS, BN,VB, and SM: conceptualization. VM, SM, SB, SP, AI, VB, and LB: methodology. SM: statistical analysis. VM, VB, SM, FE, CM, GG, and SP: investigation. VB, SM, SB, and CF: resources. SM, GG, CM, and TF: data curation. VM, SM, and VB: writing—original draft preparation. VM, SM, BN, and VB: writing—review and editing. VB, BN, and SS: supervision and project administration. VB and BN: funding acquisition. All authors have read and agreed to the published version of the manuscript.

## Funding

This research project was funded by Tuscany Region (Grant No 20RSVB—PREVIEW: PRedicting the EVolution of SubjectIvE Cognitive Decline to Alzheimer's Disease With machine learning) and by RICATEN21 (Ateneo Università di Firenze, fondi Ateneo 2021).

## Conflict of Interest

The authors declare that the research was conducted in the absence of any commercial or financial relationships that could be construed as a potential conflict of interest.

## Publisher's Note

All claims expressed in this article are solely those of the authors and do not necessarily represent those of their affiliated organizations, or those of the publisher, the editors and the reviewers. Any product that may be evaluated in this article, or claim that may be made by its manufacturer, is not guaranteed or endorsed by the publisher.
